# An in vivo tumour organoid model based on the chick embryonic chorioallantoic membrane mimics key characteristics of the patient tissue: a proof-of-concept study

**DOI:** 10.1186/s13550-024-01151-0

**Published:** 2024-09-27

**Authors:** Katarína Benčurová, Loan Tran, Joachim Friske, Kajetana Bevc, Thomas H. Helbich, Marcus Hacker, Michael Bergmann, Markus Zeitlinger, Alexander Haug, Markus Mitterhauser, Gerda Egger, Theresa Balber

**Affiliations:** 1 Division of Nuclear Medicine, Department of Biomedical Imaging and Image-Guided Therapy, Medical University of Vienna, Vienna, Austria; 2 Ludwig Boltzmann Institute Applied Diagnostics, Vienna, Austria; 3 Department of Pathology, Medical University of Vienna, Vienna, Austria; 4 Division of Molecular and Structural Preclinical Imaging, Department of Biomedical Imaging and Image-Guided Therapy, Medical University of Vienna, Vienna, Austria; 5 Division of Visceral Surgery, Department of General Surgery, Medical University of Vienna, Vienna, Austria; 6 Department of Clinical Pharmacology, Medical University of Vienna, Vienna, Austria; 7 Christian Doppler Laboratory Applied Metabolomics, Vienna, Austria; 8 Department for Inorganic Chemistry, Faculty of Chemistry, University of Vienna, Vienna, Austria; 9 Joint Applied Medicinal Radiochemistry Facility of the University of Vienna and the Medical University of Vienna, Vienna, Austria; 10 Comprehensive Cancer Center, Medical University of Vienna, Vienna, Austria

**Keywords:** Patient-derived organoids, PDX, CAM, In ovo, [^68^Ga]Ga-Pentixafor, 2-[^18^F]FDG, PET/MRI, CRC

## Abstract

**Background:**

Patient-derived tumour organoids (PDOs) are highly advanced in vitro models for disease modelling, yet they lack vascularisation. To overcome this shortcoming, organoids can be inoculated onto the chorioallantoic membrane (CAM); the highly vascularised, not innervated extraembryonic membrane of fertilised chicken eggs. Therefore, we aimed to (1) establish a CAM patient-derived xenograft (PDX) model based on PDOs generated from the liver metastasis of a colorectal cancer (CRC) patient and (2) to evaluate the translational pipeline (patient – in vitro PDOs – in vivo CAM-PDX) regarding morphology, histopathology, expression of C-X-C chemokine receptor type 4 (CXCR4), and radiotracer uptake patterns.

**Results:**

The main liver metastasis of the CRC patient exhibited high 2-[^18^F]FDG uptake and moderate and focal [^68^Ga]Ga-Pentixafor accumulation in the peripheral part of the metastasis. Inoculation of PDOs derived from this region onto the CAM resulted in large, highly viable, and extensively vascularised xenografts, as demonstrated immunohistochemically and confirmed by high 2-[^18^F]FDG uptake. The xenografts showed striking histomorphological similarity to the patient’s liver metastasis. The moderate expression of CXCR4 was maintained in ovo and was concordant with the expression levels of the patient’s sample and in vitro PDOs. Following in vitro re-culturing of CAM-PDXs, growth, and [^68^Ga]Ga-Pentixafor uptake were unaltered compared to PDOs before transplantation onto the CAM. Although [^68^Ga]Ga-Pentixafor was taken up into CAM-PDXs, the uptake in the baseline and blocking group were comparable and there was only a trend towards blocking.

**Conclusions:**

We successfully established an in vivo CAM-PDX model based on CRC PDOs. The histomorphological features and target protein expression of the original patient’s tissue were mirrored in the in vitro PDOs, and particularly in the in vivo CAM-PDXs. The [^68^Ga]Ga-Pentixafor uptake patterns were comparable between in vitro, in ovo and clinical data and 2-[^18^F]FDG was avidly taken up in the patient’s liver metastasis and CAM-PDXs. We thus propose the CAM-PDX model as an alternative in vivo model with promising translational value for CRC patients.

**Graphical Abstract:**

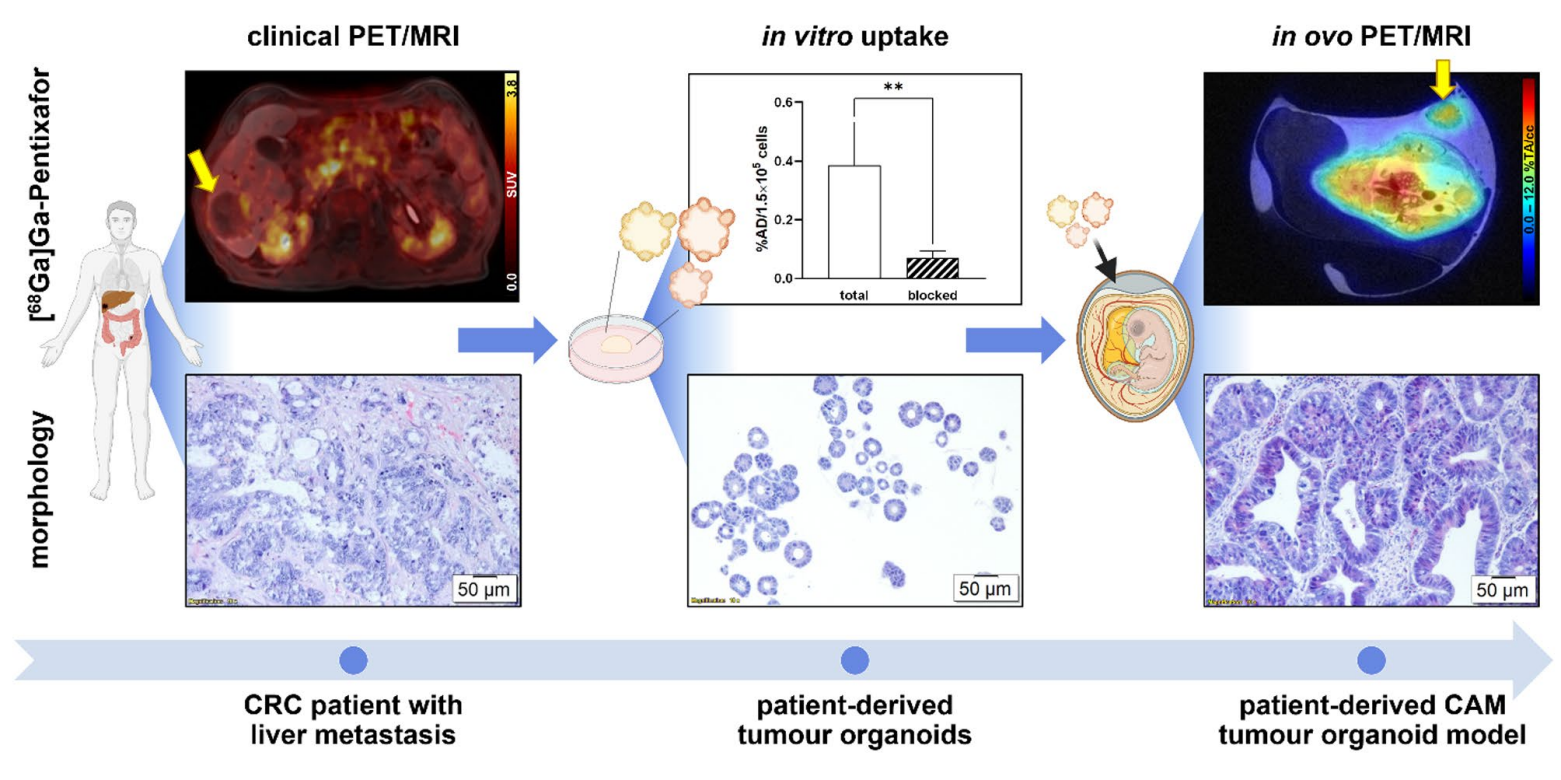

**Supplementary Information:**

The online version contains supplementary material available at 10.1186/s13550-024-01151-0.

## Background

Colorectal cancer (CRC), the third most common and the second deadliest malignancy, presents an increasingly significant public health concern [1, 2]. Metastasis to the liver dramatically reduces patient survival and both liver metastasis and survival of CRC patients have been linked to an increased expression of C-X-C chemokine receptor type 4 (CXCR4) [3, 4].

Since the creation of the “in vitro intestine” in 2009 [5], organoids have played an important role in disease modelling and drug discovery [6–8]. These self-organising and continuously expanding three-dimensional (3D) multicellular clusters recapitulate native tissue in vitro and provide a more physiologically relevant milieu compared to the well-established classical two-dimensional cell monolayers or cell line-derived 3D spheroids [6, 8, 9]. Moreover, patient-derived tumour organoids (PDOs) have emerged as a promising experimental model for personalised medicine [6, 10, 11], as they have shown high phenotypic and genotypic similarity [7, 12] as well as similar treatment response [7, 12, 13] compared to the original patient tumours. Despite being a highly advanced in vitro model, classical organoids remain incomplete as they lack the tumour microenvironment (TME) including a vascular network, which is essential for the exchange of oxygen, nutrients, and waste products, but also provides a structural template for growth [6, 8, 14, 15]. Instead, exchange occurs through slow infiltration which may affect growth and drug responses [10].

To circumvent this shortcoming, several groups have recently inoculated organoids onto the chorioallantoic membrane (CAM) of fertilised chicken eggs [16–21]. The use of this in ovo model is well documented for research in the field of tumour biology [22, 23], angiogenesis [24], and metastasis [25, 26]. The extraembryonic CAM is highly vascularised [27, 28] and the chick embryo is naturally immunodeficient until late stages of development [29, 30], making the model ideal for the growth of cancer specimens. While the exact onset of pain perception in the developing chick embryo is unknown, the extraembryonic CAM is not innervated and manipulations on the CAM are well tolerated [28, 31]. Experiments using fertilised chicken eggs are an ethically accepted alternative in line with the 3Rs of animal experimentation [32] and in many countries, including Austria, ethical approval is not required for the use of non-mammalian embryos [33].

Furthermore, CAM vessels are well suited for radiotracer injection, and the model has recently gained interest in the radiopharmaceutical community as an intermediate model between in vitro and in vivo testing using murine models. Over the past decade, various studies using different radiotracers, cancer models, imaging modalities, and comparisons with the mouse model have been carried out [34–45]. Recently, we used the CAM model for an initial evaluation of the CXCR4-targeting positron emission tomography (PET) tracer, [^68^Ga]Ga-Pentixafor, for CRC imaging [42]. Here, we wanted to generate a CAM patient-derived xenograft (PDX) model aiming for a more physiological representation of the patient’s characteristics in comparison to currently available in vitro models. Specific objectives of the presented work were (1) the establishment of a CAM-PDX model based on PDOs derived from a liver metastasis of a CRC patient and (2) the evaluation of the presented translational pipeline (patient – in vitro PDOs – in vivo CAM-PDX) regarding morphology, histopathology, expression of CXCR4, and radiotracer uptake patterns.

## Methods

**Fig. 1:**
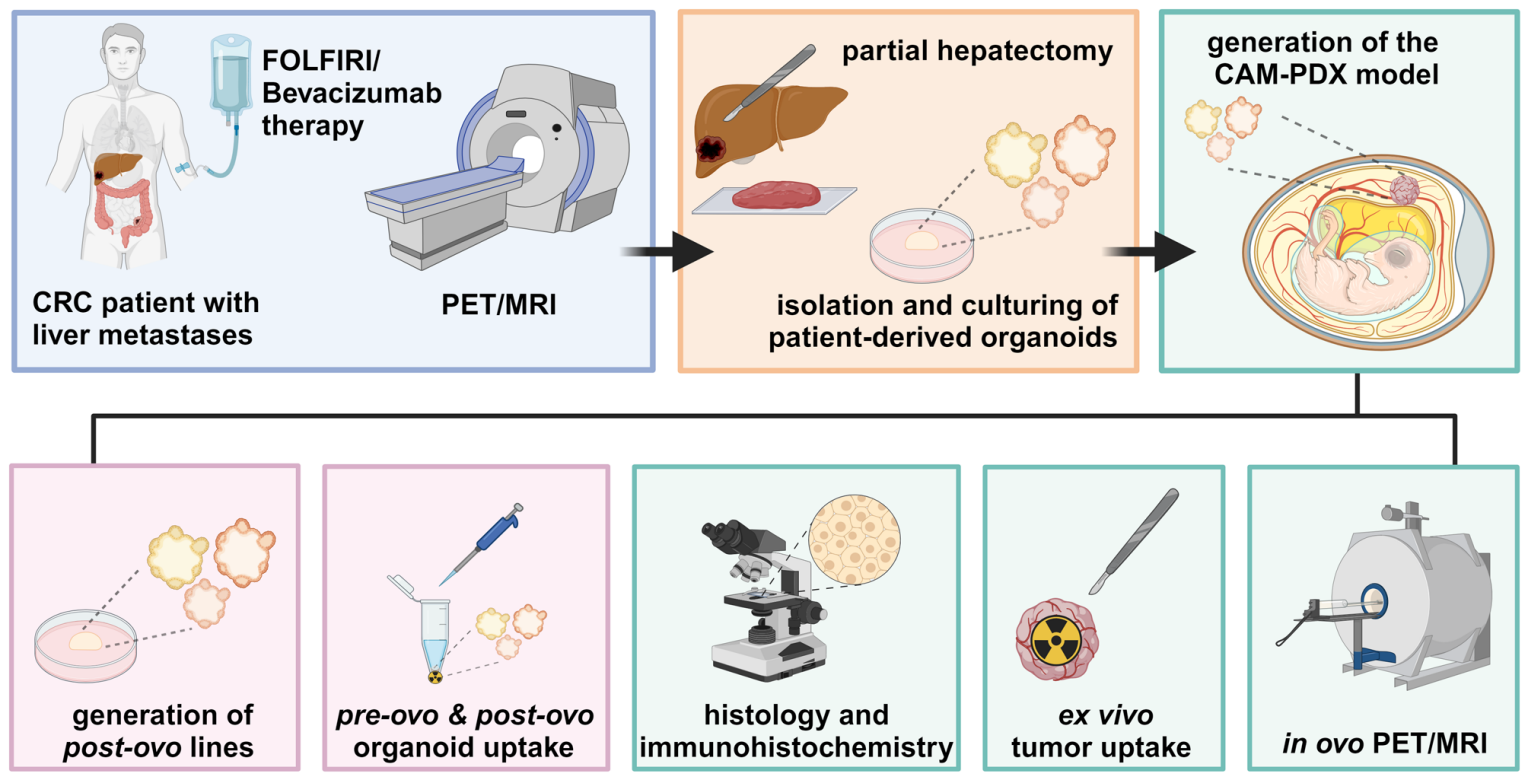
Experimental workflow of the study. Created with BioRender.com

The experimental workflow of this study is illustrated in Fig. 1.

### Radiolabelling

2-deoxy-2-[^18^F]fluoro-D-glucose (2-[^18^F]FDG) and [^68^Ga]Ga-Pentixafor were prepared for routine diagnostics following standard procedures using fully automated cassette-based synthesisers (FASTlab™; GE Healthcare, Uppsala, Sweden, and SCINTOMICS Molecular; Applied Theranostics Technologies GmbH, Fürstenfeldbruck, Germany) [46]. Quality control was performed according to the European Pharmacopoeia. To meet the higher molar activity and activity concentration required for preclinical experiments, the radiosynthesis of [^68^Ga]Ga-Pentixafor was modified for in ovo experiments and performed as published previously [42].

### Patient and clinical PET/MRI

A 78-year-old male patient, diagnosed with metastasised CRC in the liver, had previously undergone neoadjuvant therapy with FOLFIRI (FOL – folinic acid, F – 5-fluorouracil, IRI – irinotecan) and Bevacizumab. PET/magnetic resonance imaging (MRI) examinations were conducted using a Siemens Biograph mMR PET/MRI system (Siemens Healthineers, Erlangen, Germany), covering the body from the vertex to the upper thigh. Imaging using [^68^Ga]Ga-Pentixafor and 2-[^18^F]FDG was performed on two consecutive days. [^68^Ga]Ga-Pentixafor PET was acquired 40 min post-injection (*p.i.*) of 158 MBq. 2-[^18^F]FDG imaging was performed 105 min after intravenous administration of 198 MBq. The MRI protocol included among others a T_1_-weighted, two-point Dixon, 3D volume-interpolated breath-hold (VIBE) sequence as published previously [46] using contrast agent Primovist^®^ (0.1 mL/kg body weight; Bayer AG, Berlin, Germany). Data were viewed and analysed using Hermes Hybrid Viewer (Hermes Medical Solutions, Stockholm, Sweden). The maximum standardised uptake values (SUV_max_) were determined to assess the tracer uptake in the main liver metastasis. One week after the PET/MR scans, a partial hepatectomy was conducted by a surgeon. Biopsy results have shown viable tumour formations along the periphery (10%) and a necrotic core.

### PDOs isolation and in vitro culturing

PDOs were isolated from a vital, 2-[^18^F]FDG-avid, marginal part of liver metastasis that expressed CXCR4. Freshly resected tumour samples were cut into small pieces using scalpels and digested for 30 min at 37 °C in Cell Recovery Solution (Corning, NY, USA) containing Rho-associated protein kinase (ROCK) inhibitor (Y-27632; MedChemExpress, Monmouth Junction, NJ, USA). The suspension was again mechanically disrupted with scalpels and pipetting. The digested tumour tissue was pressed through a cell strainer (70 μm) and washed with phosphate-buffered saline (PBS). The cells were embedded into an extracellular matrix (ECM) (growth factor reduced Geltrex™; Gibco™, Thermo Fisher Scientific Inc., Waltham, MA, USA) and covered with factor-rich organoid ENAS (E – epidermal growth factor, N – Noggin, A – A83-01, S – SB202190) medium as detailed by others [47, 48]. PDOs were maintained in a humidified incubator at 37 °C with an atmosphere of 95% air and 5% CO_2_ and cultured in 6-well plates in droplets of 30 µL ECM (approx. 1 × 10^3^ cells/1 µL seeded) overlayed with ENAS medium for 2–4 weeks in order to obtain the required cell amounts for in ovo or in vitro experiments. PDOs were split 1–3 times a week (approx. 2 h) using TrypLE™ Express (Gibco™, Thermo Fisher Scientific Inc., Waltham, MA, USA) and the medium was exchanged every second to third day.

### Generation of the CAM-PDX model

The incubation and handling of fertilised chicken eggs were performed as previously published [42]. Eggs were incubated at 37 °C and 65% relative humidity, starting on embryo development day (EDD) 1. On EDD3, a small hole was created, which was enlarged on EDD5. Two days prior to inoculation, 1 × 10^5^ PDOs were mixed with 30 µL growth factor reduced Geltrex™ and seeded into a cell culture dish. After slight laceration of the CAM using a cotton swab, a silicone O-ring (MVQ material, 6 × 0.5 mm; Arcus, Hamburg, Germany) was positioned at the junction of two CAM vessels. Using a disinfected spatula, the PDO-ECM plugs were inoculated onto the chick embryonic CAM on EDD9. All experimental procedures were completed by EDD18. Tumours were either preserved for further histopathological evaluation or the grafts were re-cultured to generate post-ovo PDO lines (*n* = 3). Tumour volumes (V) were calculated using the formula for ellipsoid shape V = 4/3 × π × ((l × w × h)/8) with l = length, w = width, h = height [36].

### Histopathological characterisation

The harvested xenografts, PDOs, and the patient’s biopsy tissue were fixed in paraformaldehyde and embedded in paraffin. 2-µm-thick xenograft sections were subjected to haematoxylin-eosin (H&E) staining (*n* = 9), periodic acid-Schiff (PAS) staining (*n* = 3), or immunohistochemical (IHC) analyses (*n* ≥ 3 per marker) following standard procedures. IHC for Ki67 and Cytokeratin (CK) AE1/AE3 was automatically performed using an auto-stainer (VENTANA BenchMark ULTRA; Roche Tissue Diagnostics, Oro Valley, AZ, USA), while staining against CXCR4 was performed manually. Complementary histopathological analysis of PDOs and the patient’s liver metastasis was performed *n* = 1 for each staining. An additional IHC was performed for CAM-PDX samples for Desmin and cleaved Caspase 3 (CC3). The following antibodies were employed for IHC: antibody against CC3 (*n* = 5; 1:2000, clone 5A1E, no.: #9664; Cell Signaling Technology, Danvers, MA, USA), CXCR4 (*n* = 5; 1:400, clone UMB2, no.: ab12824; Abcam, Cambridge, UK), CK clone AE1/AE3 (*n* = 3; 1:100, no.: M3515; Dako, Agilent, Santa Clara, CA, USA), Desmin (*n* = 4; 1:100, clone 33, no.: M0760; Dako, Agilent, Santa Clara, CA, USA) and Ki67 (*n* = 3; ready-to-use, clone: 30 − 9, no.: 790–4286; Roche Tissue Diagnostics, Oro Valley, AZ, USA).

### CAM-PDX re-culturing

The xenografts were harvested and processed either fresh or on the following day after storage in ENAS medium at 4 °C. Corresponding to the PDOs isolation procedure, xenografts were dissected into small pieces with scalpels and digested in ROCK-inhibitor-containing Cell Recovery Solution at 37 °C for 30 min. After further mechanical disruption, the digested xenograft tissue was pressed through a 70 μm cell strainer and washed with PBS. The cells were embedded in ECM and covered with ENAS medium. The post-ovo PDO lines were cultured according to the PDO culture procedures described above.

### In vitro [^68^Ga]Ga-Pentixafor uptake in PDOs

Seven days prior to the experiment, 3.5 × 10^5^ cells per PDO line were seeded (1 × 10^4^ cells/1 µL growth factor reduced Geltrex™). ENAS medium was exchanged every other day. On the experimental day, cells were recovered from the ECM using Cell Recovery Solution (40 min, 150 rpm, on ice). After centrifugation and washing with Dulbecco’s phosphate-buffered saline (DPBS), PDO pellets were resuspended in the ENAS medium. The PDO suspension was aliquoted into Protein LoBind^®^ Tubes (Eppendorf SE, Hamburg, Germany). For assessing [^68^Ga]Ga-Pentixafor binding specificity, CXCR4 antagonist I AMD3100 (Calbiochem^®^, Merck KGaA, Darmstadt, Germany) (1 µM final concentration; blocked uptake) or ultra-pure water (vehicle control; total uptake) were added to each tube, followed by 7 pmol [^68^Ga]Ga-Pentixafor (138 ± 24 kBq, 14 nM final concentration). Three tubes were prepared as a reference to determine the applied dose (AD). Three additional tubes were treated analogously to evaluate the unspecific binding to tube plastic. Cells (and control tubes) were incubated with the radiotracer for 2 h at 37 °C with gentle shaking (300 rpm). Two additional tubes were counted to estimate cell numbers. After incubation, cells were centrifuged, and the supernatant was collected (supernatant fraction). The PDOs were washed twice with ice-cold DPBS to obtain the wash fraction. Medium was added to the PDO pellet (cell fraction) and all fractions and references were gamma counted (2480 Wizzard^2^^®^; PerkinElmer, Waltham, MA, USA). Cell uptake was calculated as the percentage of applied dose per 1.5 × 10^5^ cells (%AD/1.5 × 10^5^ cells). The experiments (*n* = 5) were performed in triplicates with the PDOs before inoculation onto the CAM (pre-ovo) and compared to three different post-ovo PDO lines (*n* = 2–3 per post-ovo line, data grouped).

### Ex vivo gamma counting

A dose of 2.80 ± 0.9 MBq 2-[^18^F]FDG (*n* = 3) or 8.35 ± 10.2 MBq [^68^Ga]Ga-Pentixafor (≥ 96% radiochemical purity; 1.53 ± 1.6 nmol; *n* = 3 baseline, *n* = 3 blocking) was injected on EDD16 or EDD17 into a CAM vessel as published previously [42]. The eggs of the [^68^Ga]Ga-Pentixafor blocking group were additionally co-injected with 315 µg AMD3100 (in 50 µL; approx. 15 mg/kg body weight when assuming an embryo weight of 21 g [49]). Subsequently, the eggs were incubated at 37 °C for 48 min to allow for the distribution of the radiotracer. Prior to decapitation (62 min *p.i.*) and tumour removal, anaesthesia was induced with isoflurane (6 min, 3% in 2 L air/min). Samples were weighed and gamma counted (Hidex Automatic Gamma Counter, Mainz, Germany) and radiotracer uptake was calculated as the percentage of injected dose (ID) per gram (%ID/g).

### Preclinical PET/MRI

In addition to the ex vivo assessment of xenograft uptake, PET/MRI was performed as previously published [42] to visualise radiotracer uptake in a separate group of eggs (*n* = 1–3 per group). PET was measured simultaneously with MR using a preclinical MRI scanner (BioSpec^®^ 94/30 with 9.4 Tesla; Bruker Biospin, Ettlingen, Germany) with a dedicated PET insert (model Si 168; Bruker Biospin, Ettlingen, Germany) and an 86 mm PET-compatible coil (model T20202V3; Bruker Biospin, Ettlingen, Germany). Paravision 360 V3.2 software (Bruker Biospin, Ettlingen, Germany) was used for data acquisition.

7.14 ± 5.0 MBq [^68^Ga]Ga-Pentixafor (≥ 97% radiochemical purity, 1.38 ± 0.8 nmol) was injected into a CAM vessel on EDD16 (baseline, *n* = 3) and EDD17 (blocking, *n* = 1), followed by 10.02 MBq 2-[^18^F]FDG on EDD18 (*n* = 1). Blocking was performed as described for ex vivo analysis. The eggs were incubated at 37 °C to allow the radiotracer to distribute and embryos were subsequently anaesthetised with 3% isoflurane in 2 L air/min for 6 min before the scan start. Anaesthesia was maintained throughout the whole duration of the scan (2% isoflurane). Static PET scan (15 min) was started 60 min *p.i*. For the generation of attenuation maps, a 3D T_1_-weighted iso-voxel fast low-angle shot (FLASH) sequence covering the whole egg in isotropic resolution was acquired. T_2_-weighted rapid acquisition with relaxation enhancement (TurboRARE) sequences (axial, coronal) were acquired as an anatomical reference. Sequence details can be found elsewhere [42]. PET data were corrected for scatter, deadtime, and random coincidences, and attenuation correction for the egg, cradle, and MR coil was applied. List-mode data were reconstructed using the maximum-likelihood expectation-maximisation algorithm (18 iterations, 0.5 × 0.5 × 0.5 mm pixel size, 180 × 180 × 300 matrix size). Image datasets were analysed using the PMOD software version 3.807 (PMOD Technologies, Zürich, Switzerland). Xenografts were delineated based on the PET information and the delineation was controlled with anatomical MR information. A sphere was placed around the whole egg to extract the total injected activity (TA) to determine the percentage of total activity per cm^3^ (%TA/cc).

### Statistics

Statistical analyses and graph preparations were performed using GraphPad Prism software version 8.2.1 (GraphPad Software, Inc., San Diego, CA, USA). Data were tested for normal distribution and paired or unpaired *t*-test was used for group comparison as indicated. A p-value < 0.05 was set as the significance threshold. The reported values represent mean ± standard deviation (SD).

## Results

### Clinical PET/MRI

**Fig. 2:**
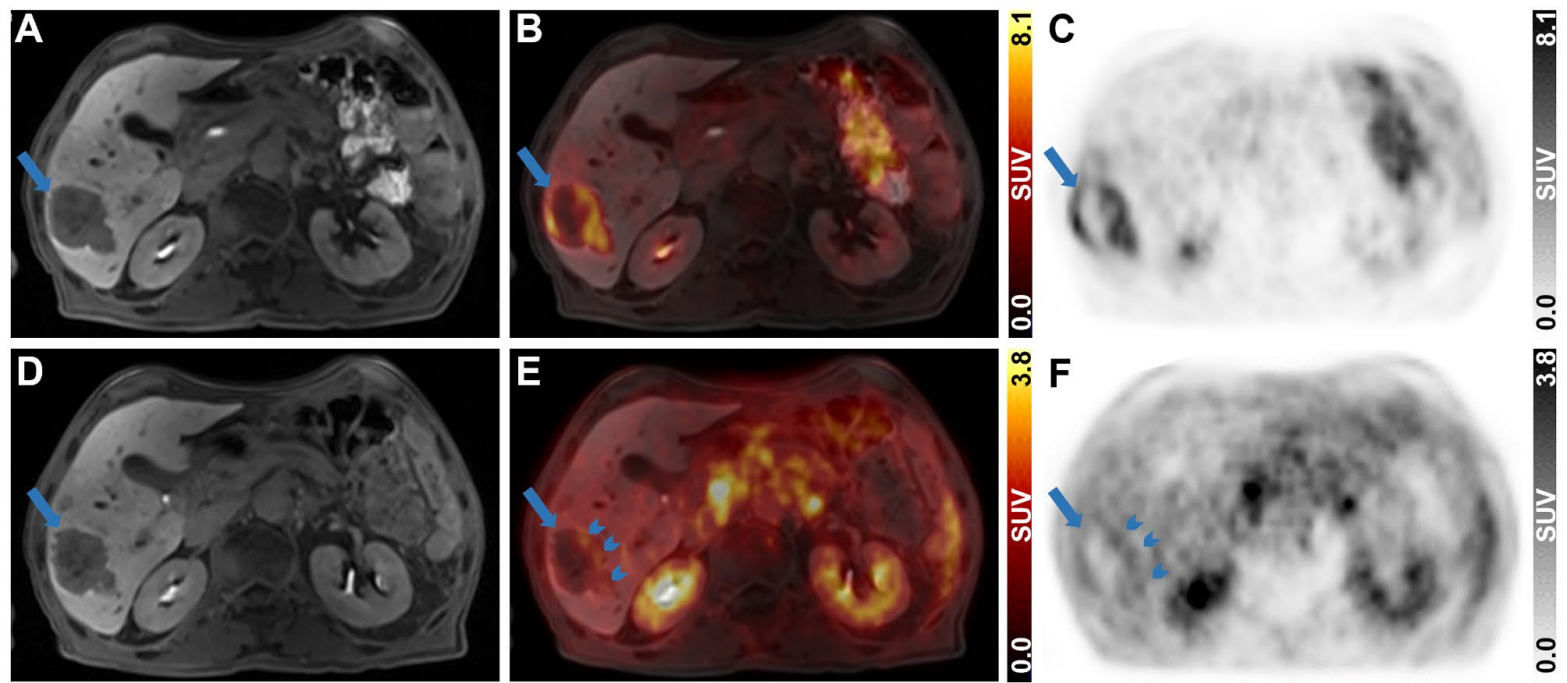
Diagnostic abdominal 2-[^18^F]FDG and [^68^Ga]Ga-Pentixafor PET/MR images of the liver metastasis in the CRC patient. (**A**, **D**) Contrast-enhanced T_1_-VIBE MR images, (**C**, **F**) PET images and (**B**, **E**) fused PET and MR images are presented in axial views for 2-[^18^F]FDG (105 min *p.i.*, 198 MBq, upper row) and [^68^Ga]Ga-Pentixafor (40 min *p. i.*, 158 MBq, lower row). Long blue arrows indicate the liver metastasis and short blue arrows indicate focal [^68^Ga]Ga-Pentixafor uptake

A patient diagnosed with CRC exhibiting multiple liver metastases underwent diagnostic PET/MRI post-neoadjuvant therapy. The primary liver metastasis (Fig. 2A, D) was centrally necrotic and accumulated 2-[^18^F]FDG at the periphery (SUV_max_ = 8.1) (Fig. 2B, C). [^68^Ga]Ga-Pentixafor uptake was focal and peripheral with an SUV_max_ of 2.8 (Fig. 2E, F).

### Generation of the CAM-PDX model and its histopathological characterisation

Liver metastasis organoids derived from the CRC patient, inoculated as pre-formed organoids, were successfully grown on the CAM, generating large, viable, and vascularised CAM-PDXs. The survival rate of the embryos after PDO inoculation was 64% (18/28). Ten embryos died before the commencement of in vivo imaging/ex vivo gamma counting: three on EDD10-11, four on EDD12-14, and three on EDD15-16. The tumour take rate was 89% (16/18). Within 7–9 days after inoculation of 1 × 10^5^ PDOs (EDD16–EDD18), grafts with a volume of 42.53 ± 30.1 mm^3^ (range: 13.5–140.7 mm^3^, *n* = 16) were obtained (Supplementary Fig. 1).

**Fig. 3:**
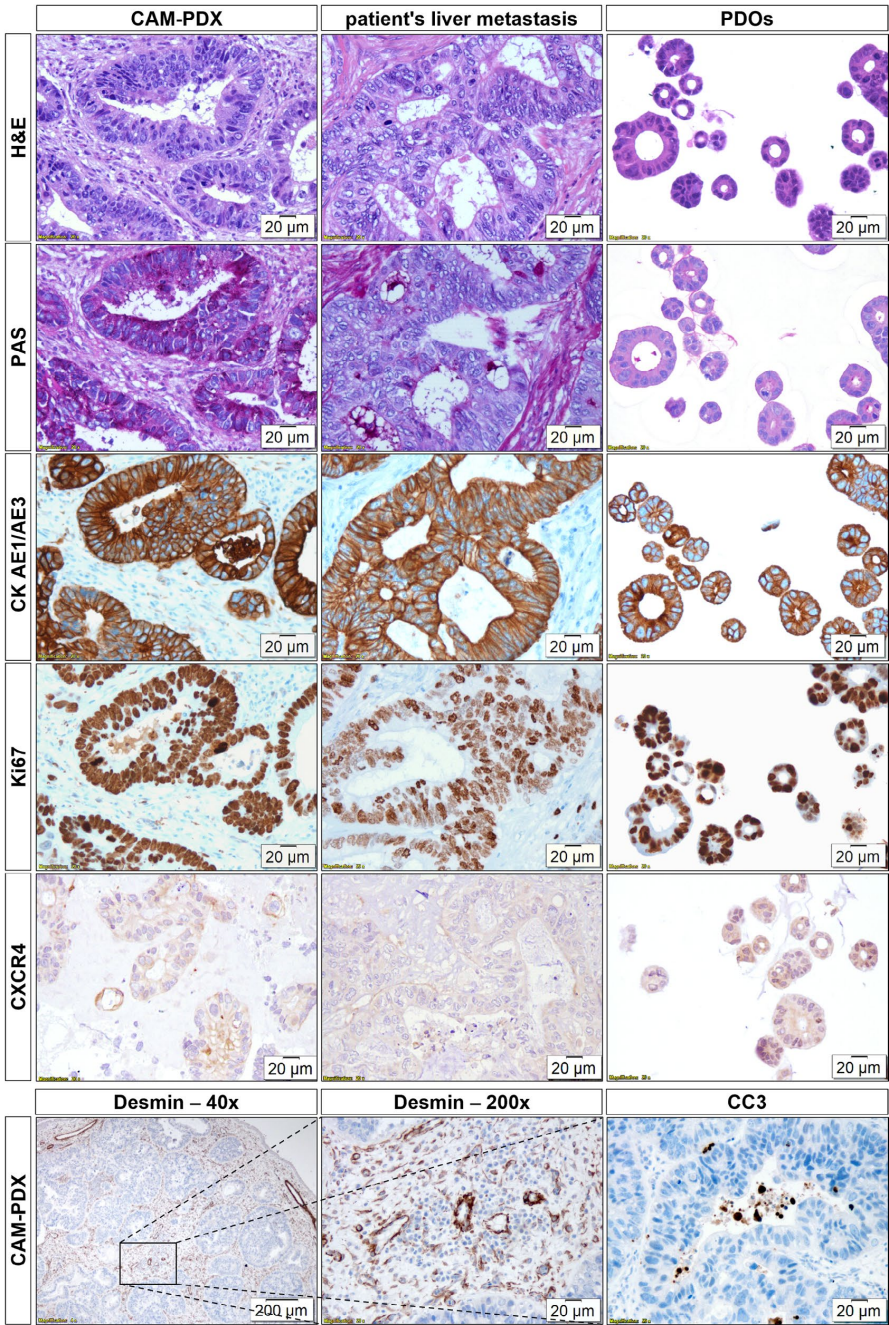
Histopathological characterisation of CAM-PDXs and comparison with the patient’s liver metastasis and in vitro PDOs. H&E, PAS stain, and IHC for CK AE1/AE3, Ki67, and CXCR4 are shown for CAM-PDXs (*n* ≥ 3), patient’s liver metastasis (*n* = 1), and PDOs (*n* = 1). Additional IHC for CAM-PDXs for Desmin and CC3 (*n* ≥ 4) is shown. Magnification: 200-fold; 40-fold (Desmin IHC (left)). The 200x Desmin image corresponds to the central part of the 40x magnification as indicated by a black rectangle

Histopathological characterisation of CAM-PDXs in comparison with the patient’s liver metastasis and the in vitro PDOs is shown in Fig. 3. Growth of CRC PDOs on the CAM led to the formation of glandular structures with hyperchromatic tumour cell nuclei and prominent nucleoli strongly resembling the patient’s liver metastasis. The stromal components of the CAM morphologically resembled the TME of the patient. Tumour cells within the CAM-PDXs showed strong staining for PAS, especially on their luminal side, indicating mucus production, similar to the metastatic tissue, while in vitro PDOs were largely PAS-negative. Human carcinoma cells can be distinguished from the connective tissue using CK AE1/AE3. Tumour cells in the CAM-PDXs, the patient’s sample, and the PDOs show a highly comparable, strongly positive membrane staining. In all samples, most tumour cells were in a proliferative state, as evidenced by positive staining with an antibody against Ki67. In addition, CXCR4 staining was comparable in CAM-PDXs and metastatic tissue showing mainly membrane and weaker cytoplasmic staining, whereas in vitro PDOs displayed comparable levels of cytoplasmic and membrane staining. Macroscopic and microscopic examination of CAM-PDXs revealed a high level of vascularisation as visualised by Desmin staining. Whilst large blood vessels were primarily located at the xenografts’ periphery, smaller capillaries were interspersed between the glands. Apoptotic cells were mainly observed within the lumens of the glands as evidenced by CC3-positive cells.

### CAM-PDX re-culturing and in vitro [^68^Ga]Ga-Pentixafor uptake in PDOs

**Fig. 4:**
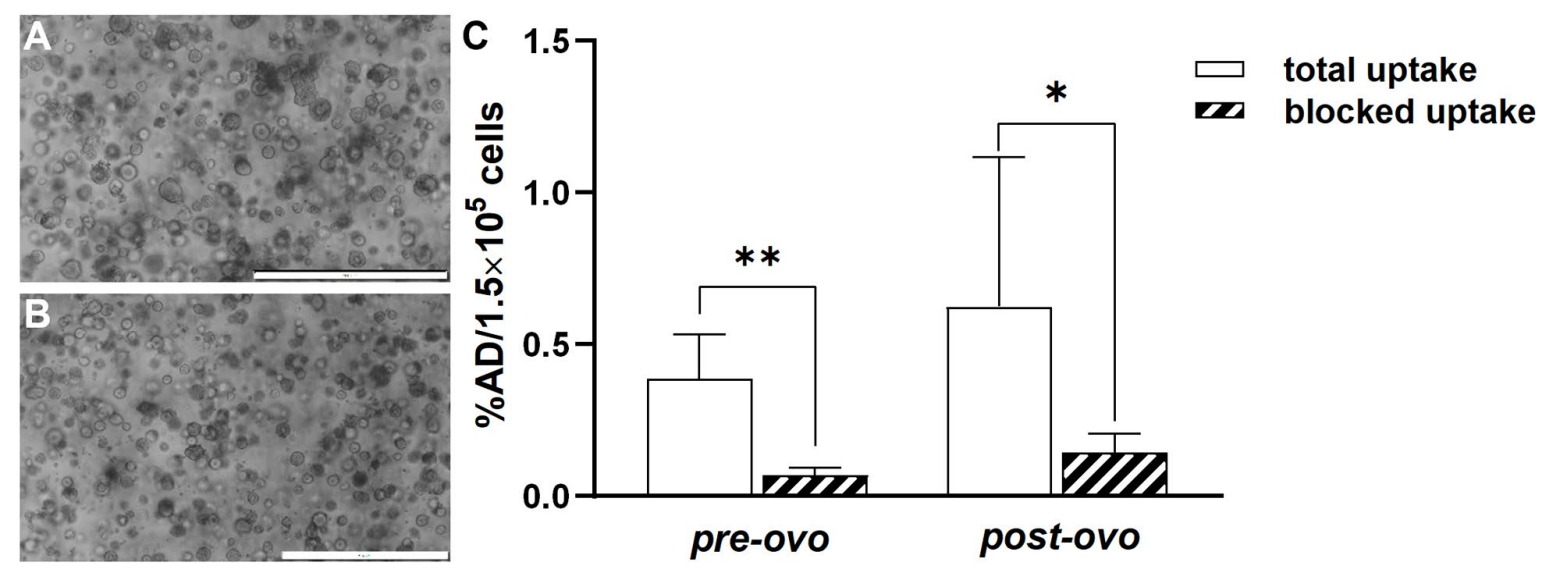
PDOs in culture and [^68^Ga]Ga-Pentixafor PDO uptake. Representative bright-field microscopic images of (**A**) *pre-ovo* and (**B**) post-ovo PDOs. Magnification: 40-fold; scale bars correspond to 500 μm. (**C**) [^68^Ga]Ga-Pentixafor uptake into pre-ovo PDOs compared to post-ovo PDOs shown as a percentage of applied dose per 1.5 × 10^5^ cells (%AD/1.5 × 10^5^ cells). Significantly different datasets are marked with an asterisk (* *p* < 0.05, ** *p* < 0.01)

Re-culturing CAM-PDXs resulted in viable post-ovo PDO lines (*n* = 3, Fig. 4B) exhibiting morphology and growth characteristics similar to the original PDO line (Fig. 4A). [^68^Ga]Pentixafor was moderately taken up in both pre-ovo and post-ovo PDOs (Fig. 4C, Supplementary Table 1). This uptake was significantly reduced when co-incubating with a 1000-fold excess of CXCR4 antagonist in pre-ovo (*p* = 0.0077, 82% blocking) and post-ovo PDOs (*p* = 0.0289, 77% blocking, paired *t*-test) proving specific binding of [^68^Ga]Ga-Pentixafor. No significant differences in total (*p* = 0.3253) or specific uptake (*p* = 0.45) were observed between pre-ovo and post-ovo PDOs (*n* = 5, unpaired *t*-test).

### Ex vivo gamma counting

[^68^Ga]Ga-Pentixafor and 2-[^18^F]FDG uptake into CAM-PDXs were investigated by ex vivo gamma counting. Despite the notable accumulation of [^68^Ga]Ga-Pentixafor in CAM-PDXs, there was only a trend towards blocking, but no significant difference compared to the blocking group (11.23 ± 2.7 vs. 8.91 ± 2.1%ID/g, *p* = 0.3047, unpaired *t*-test, *n* = 3). 2-[^18^F]FDG was avidly taken up in CAM xenografts derived from CRC PDOs (10.51 ± 2.0%ID/g, *n* = 3) (Supplementary Fig. 2, Supplementary Table 2).

### Preclinical PET/MRI

**Fig. 5:**
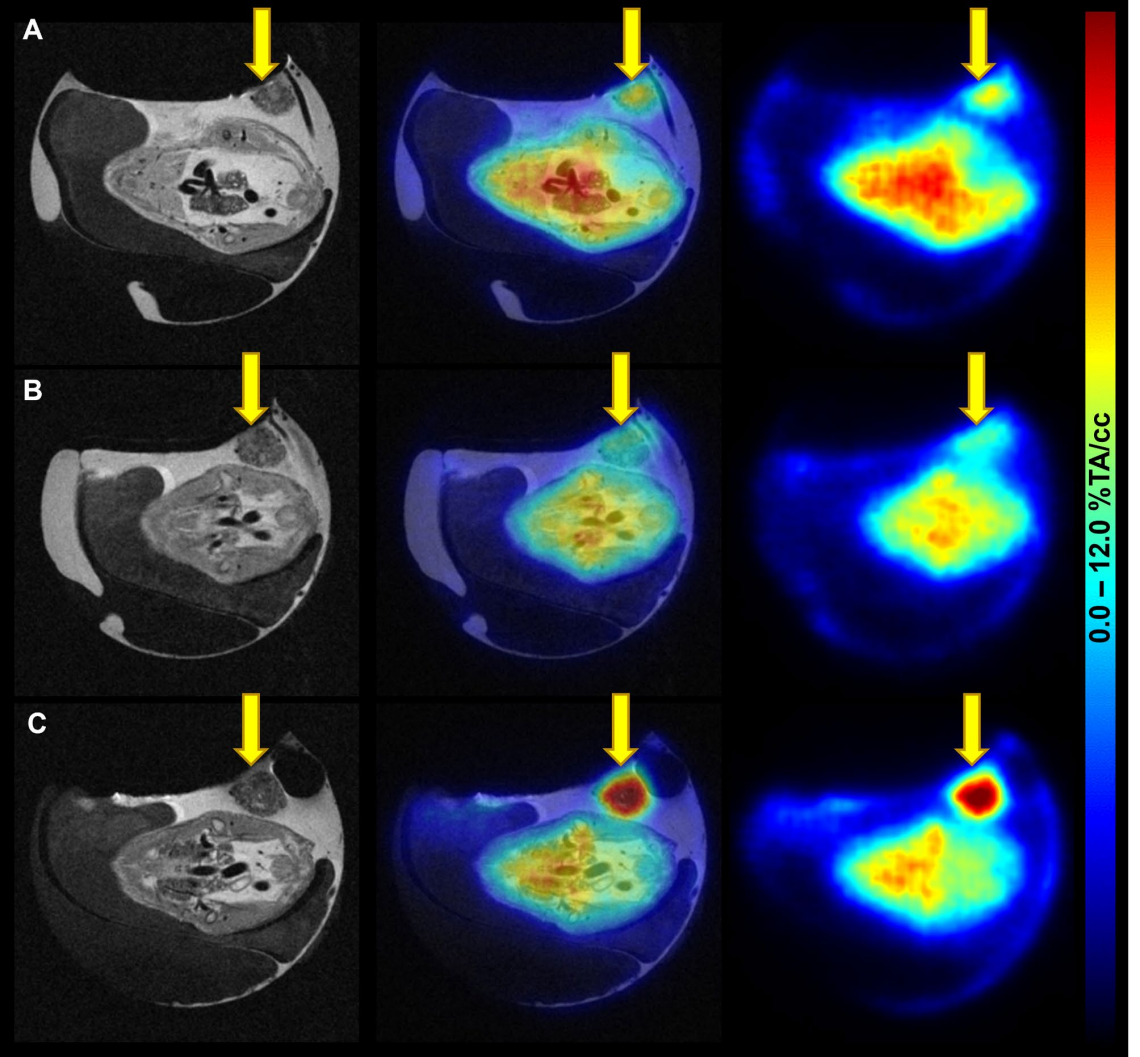
Images of [^68^Ga]Ga-Pentixafor and 2-[^18^F]FDG PET/MRI in the CAM-PDX model of CRC. (**A**) [^68^Ga]Ga-Pentixafor baseline (EDD16; 4.1 MBq, 1.6 nmol [^68^Ga]Ga-Pentixafor), (**B**) blocking (EDD17; 7.2 MBq, 2.3 nmol [^68^Ga]Ga-Pentixafor; 315 µg AMD3100), and (**C**) 2-[^18^F]FDG scans (EDD18; 5.8 MBq) were performed using the same subject on three consecutive days. Left: T_2_-TurboRARE MR images, right: PET images 60 min *p.i.*, middle: fused PET/MR images in axial view. PET data are provided as a percentage of total activity per cm^3^ (%TA/cc). The xenograft is marked with yellow arrows

To additionally visualise tumour uptake, [^68^Ga]Ga-Pentixafor baseline and blocking scans as well as the 2-[^18^F]FDG scan were performed using the same egg on three consecutive days. Both tracers delivered high image contrast and tumours could be easily delineated. This longitudinal imaging suggests a weak specific uptake of [^68^Ga]Ga-Pentixafor into CAM-PDXs (6.64 and 5.25%TA/cc for baseline and blocking, respectively, *n* = 1). The T_2_-weighted MR images provide additional insight into the development and location of the xenograft (Fig. 5). Quantification of the complete imaging data can be found in Supplementary Table 2.

The injection success rate was 82% (14/17). One PET/MRI scan and two ex vivo gamma counted eggs had to be excluded from the analysis due to failed injection. Two additional eggs could not be injected and were therefore not imaged/gamma counted.

## Discussion

This work reports the successful establishment of a CAM-PDX model derived from liver metastasis organoids from a CRC patient highly recapitulating the patient’s characteristics: The morphology, histopathology, and CXCR4 expression of the patient’s liver metastasis were reflected in in vitro PDOs and especially in CAM xenografts underlining the translational value of this alternative in vivo model for CRC patients. Moreover, 2-[^18^F]FDG and [^68^Ga]Ga-Pentixafor uptake patterns were comparable between CAM-xenografts and the patient’s liver metastasis.

Previously, various organoids have been grown on the chick embryonic CAM employing different inoculation strategies and techniques leading to enhanced maturation of the implanted organoids as compared to their in vitro counterparts [16, 17, 19–21]. In this study, grafting small PDOs grown for 2 days from single cells resulted in xenografts resembling gland-forming CRC adenocarcinoma with moderate architectural complexity. Importantly, the morphology of the in ovo xenografts matched the morphology of the CRC patient’s liver metastasis and glandular formations of the in vitro PDOs.

Induction of angiogenesis is one of the hallmarks of cancer [50], but is naturally absent in in vitro models. Vascularisation of CAM xenografts derived from cells [51, 52], tissues [53], and organoids [16–18, 20, 21] has been reported in the literature, and vessels generated in vitro were shown to connect to CAM vessels [14, 20, 21]. Using IHC, we demonstrated extensive vascularisation of the CAM-PDXs which was further confirmed by radiotracer uptake into the grafts. The high 2-[^18^F]FDG accumulation (mean uptake 10.5%ID/g) is in line with the high uptake in the viable periphery of the patient’s liver metastasis (SUV_max_ = 8.1) and additionally demonstrates glucose turnover in the grafts and confirms their high viability. CC3 staining, performed to assess the extent of apoptosis in the CAM-PDX model, revealed a low number of CC3-positive cells localised mainly in the lumens of the glands, further confirming the high viability of the grafts. The CAM-PDXs consist not only of tumour cells and blood vessels but also of CAM-connective tissue, as indicated by histological analyses. The visual morphological similarity of this CAM-derived stroma with the TME of the patient’s liver metastasis is striking.

Additionally, three CAM xenografts were harvested and could be re-cultured, yielding post-ovo PDO lines similar in growth and morphology to the PDOs before grafting onto the CAM. There was no significant difference in [^68^Ga]Ga-Pentixafor uptake between the pre-ovo and post-ovo PDOs suggesting that growth on the CAM does not alter these phenotypical characteristics. Moreover, tracer uptake was significantly reduced by 82% and 77% in both pre-ovo and post-ovo PDOs, respectively, when co-incubated with an excess of the CXCR4 antagonist, proving specific binding in vitro. Of note, there are not many studies investigating radiotracer uptake in organoids [54, 55]. The modest but specific uptake in the PDOs corresponds to the low uptake in the patient’s liver metastasis, suggesting preservation of the weak CXCR4 expression after growth in ovo. This was confirmed by IHC and is consistent with CXCR4 expression in the patient’s liver metastasis and PDOs. CXCR4 staining was observed on the cell membrane and to varying extent also in cytoplasm of all samples, which is concordant with the literature [56].

[^68^Ga]Ga-Pentixafor PET/MRI was performed under baseline and blocking conditions in the same egg on two subsequent days suggesting a weak specific uptake into CAM-PDXs. However, longitudinal studies with repeated injections are extremely challenging as the blood loss due to injection often leads to embryo death. This was also the case in the present study and resulted in a reduced sample size. Albeit, ex vivo assessment of [^68^Ga]Ga-Pentixafor uptake in CAM-PDXs did not reveal a significant difference between the baseline and blocking group (mean uptake 11.23 vs. 8.91%ID/g), possibly due to the low CXCR4 expression combined with a small amount of inoculated cells (1 × 10^5^ PDOs) and a natural egg-to-egg variation. While this matches the low uptake in the patient’s liver metastasis (SUV_max_ = 2.8), the isolated in vitro system can show the low specific binding which the complex in ovo model cannot. Furthermore, the herein-observed differences in [^68^Ga]Ga-Pentixafor uptake values as quantified based on imaging vs. ex vivo gamma counting can be explained with the long positron range of Ga-68 as already reported elsewhere [57, 58].

This preliminary study presents a translational pipeline transitioning from clinical imaging to PDOs and culminating in the in ovo model, which enables rapid growth and vascularisation of PDOs. To the best of our knowledge, this is the first study of its kind in a nuclear medicine context. The presented CAM-PDX model is a physiological and translational in vivo model that complies with the 3Rs of animal experimentation [32] and is of particular interest to radiopharmaceutical departments or imaging facilities without animal housing or animal ethics approval. Since the oxygen and nutrients are supplied by the developing blood vessels, tumours develop in general much faster on the CAM compared to immunodeficient mice (3–4 days vs. 3–4 weeks for cell lines) [9].

The small sample size for ex vivo assessment, but especially the low number of individuals imaged with PET/MRI, are the main limitations of this study. Furthermore, the use of only one PDO line derived from a single patient limits the generalisability of these findings. Nevertheless, we have proven the concept for the inoculation of patient-derived organoids onto chick embryonic CAM and believe that this model could serve as a patient avatar for drug testing in a personalised manner in the future, pending further studies with more PDO lines and a comparison of response to treatment.

## Conclusions

This study proves the successful inoculation of PDOs onto the chick embryonic CAM, resulting in viable and vascularised grafts within a short time. Importantly, histomorphology, target protein expression, and tracer uptake patterns were conserved between the original tumour tissue and the CAM-PDXs, suggesting that organoid-derived CAM models can reflect the patient tumour phenotype and molecular characteristics.

## Electronic supplementary material

Below is the link to the electronic supplementary material.

Supplementary Material 1

## Data Availability

The datasets generated during and/or analysed during the current study are available from the corresponding author on reasonable request.

## Abbreviations

%AD/1.5 × 10^5^ cells: Percentage of applied dose per 1.5 × 10^5^ cells
%ID/g: Percentage of injected dose per gram
%TA/cc: Percentage of total activity per cm^3^
2-[^18^F]FDG: 2-deoxy-2-[^18^F]fluoro-D-glucose
AD: Applied dose
CAM: Chorioallantoic membrane
CC3: Cleaved Caspase 3
CK: Cytokeratin
CRC: Colorectal cancer
CXCR4: C-X-C chemokine receptor type 4
DPBS: Dulbecco’s phosphate-buffered saline
ECM: Extracellular matrix
EDD: Embryo development day
ENAS: E (epidermal growth factor), N (Noggin), A (A83-01), S (SB202190)
FLASH: Fast low-angle shot
FOLFIRI: FOL (folinic acid), F (5-fluorouracil), IRI (irinotecan)
H&E: Haematoxylin-eosin
ID: Injected dose
IHC: Immunohistochemistry
MRI: Magnetic resonance imaging
*p.i.*: Post-injection
PAS: Periodic acid-Schiff
PBS: Phosphate-buffered saline
PDOs: Patient-derived tumour organoids
PDX: Patient-derived xenograft
PET: Positron emission tomography
Post-ovo: PDO line generated after re-culturing of CAM-PDX
Pre-ovo: PDO line before inoculation onto the CAM
ROCK: Rho-associated protein kinase
SUV_max_: Maximum standardised uptake value
TA: Total activity
TME: Tumor microenvironment
TurboRARE: Rapid acquisition with relaxation enhancement
VIBE: Volume-interpolated breath-hold
