# Supplementary material for: An in vivo tumour organoid model based on the chick embryonic chorioallantoic membrane mimics key characteristics of the patient tissue: a proof-of-concept study
Source: EJNMMI Res. 2024 Sep 27;14:86. doi: 10.1186/s13550-024-01151-0 (PMC11436503; doi:10.1186/s13550-024-01151-0)
Supplement: Supplementary file 1 — Supplementary Material 1 [file 13550_2024_1151_MOESM1_ESM.docx]

**Supplementary Information**

(EJNMMI Research)

**An *in vivo* tumour organoid model based on the chick embryonic chorioallantoic membrane mimics key characteristics of the patient tissue: a proof-of-concept study**

Katarína Benčurová ^1,2^, Loan Tran ^2,3^, Joachim Friske ^4^, Kajetana Bevc ^2^, Thomas H. Helbich ^4^, Marcus Hacker ^1^, Michael Bergmann ^5^, Markus Zeitlinger ^6^, Alexander Haug ^1,7^, Markus Mitterhauser ^1,2,8,9,*^, Gerda Egger ^2,3,10^, Theresa Balber ^1,2,9^

^1^ Division of Nuclear Medicine, Department of Biomedical Imaging and Image-Guided Therapy, Medical University of Vienna, Vienna, Austria

^2^ Ludwig Boltzmann Institute Applied Diagnostics, Vienna, Austria

^3^ Department of Pathology, Medical University of Vienna, Vienna, Austria

^4^ Division of Molecular and Structural Preclinical Imaging, Department of Biomedical Imaging and Image-Guided Therapy, Medical University of Vienna, Vienna, Austria

^5^ Division of Visceral Surgery, Department of General Surgery, Medical University of Vienna, Vienna, Austria

^6^ Department of Clinical Pharmacology, Medical University of Vienna, Vienna, Austria

^7^ Christian Doppler Laboratory Applied Metabolomics, Vienna, Austria

^8^ Department for Inorganic Chemistry, Faculty of Chemistry, University of Vienna, Vienna, Austria

^9^ Joint Applied Medicinal Radiochemistry Facility of the University of Vienna and the Medical University of Vienna, Vienna, Austria

^10^ Comprehensive Cancer Center, Medical University of Vienna, Vienna, Austria

* corresponding author

**
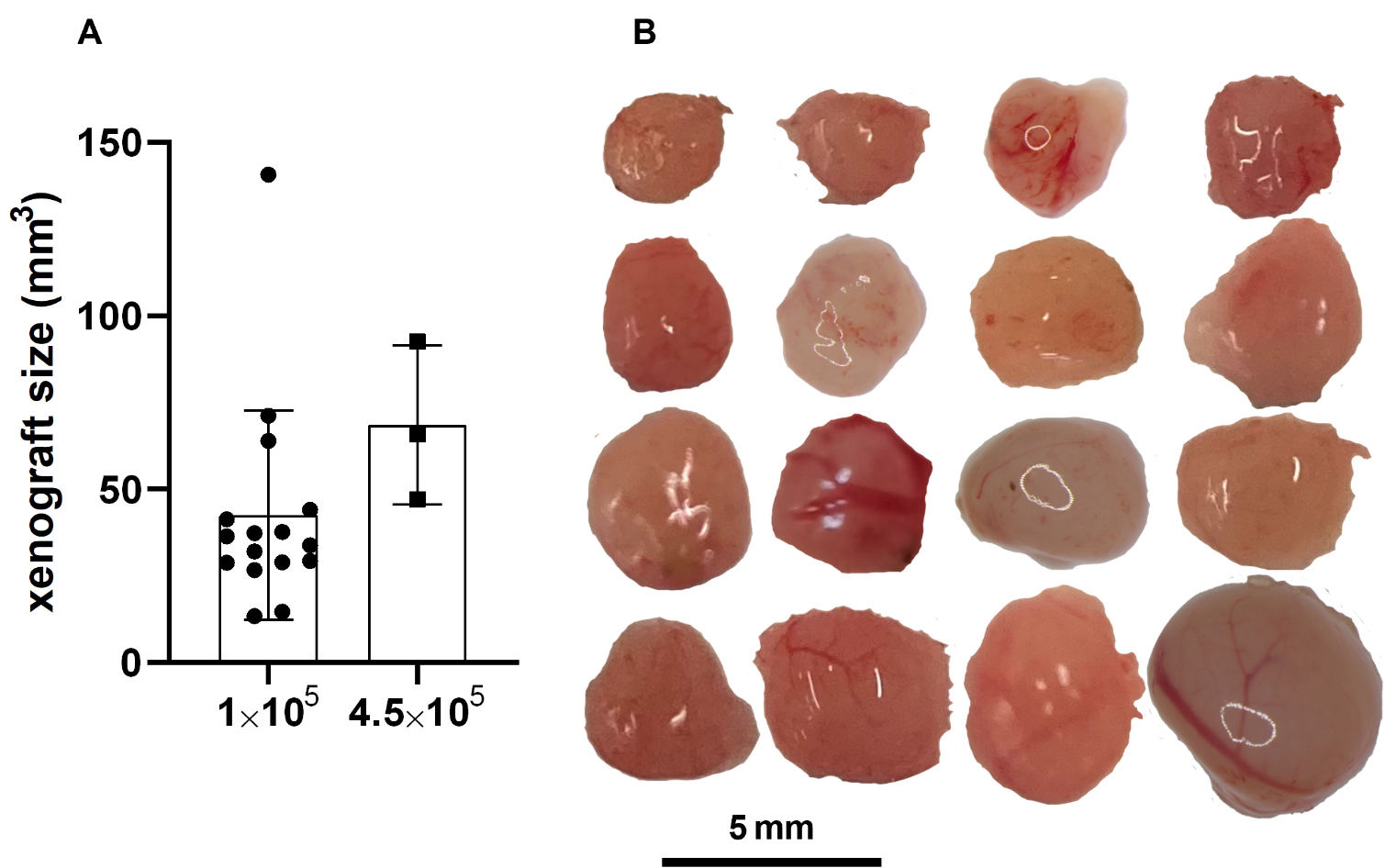
**

**Supplementary Fig. 1** Volumes and photographs of CAM-PDXs. (**A**) Size distribution of CAM-PDXs. The X-axis shows different amounts of inoculated cells and is plotted against xenograft volume in mm^3^ presented as mean ± standard deviation (SD) (Y-axis). The two groups are not significantly different (unpaired t-test, p = 0.177, n = 16 (1 × 10^5^) or n = 3 (4.5 × 10^5^)). Although there was a trend towards larger tumour volumes (68.50 ± 22.9 mm^3^) when higher cell amounts (4.5 × 10^5^) were inoculated, this approach was curtailed as it appeared to be less tolerated and resulted in lower embryo survival (50%, 3/6). (**B**) Cropped photographs of harvested CAM-PDXs (the group with inoculation of 1 × 10^5^ PDOs) are shown

**Supplementary Table 1** Uptake of [^68^Ga]Ga-Pentixafor into *pre-ovo* and *post-ovo* PDOs

|  | ***pre-ovo*** | ***post-ovo*** |
| --- | --- | --- |
| total uptake | 0.386 ± 0.15 | 0.625 ± 0.50 |
| blocked uptake | 0.069 ± 0.02 | 0.144 ± 0.06 |
| specific uptake* | 0.317 ± 0.14 | 0.481 ± 0.45 |

Uptake values were calculated as a percentage of applied dose (AD) per 1.5 × 10^5^ cells (%AD/1.5 × 10^5^ cells) and are presented as mean ± SD (n = 5 in triplicates).

^*^ specific uptake was calculated by subtracting the blocked from the total uptake

**Supplementary Fig. 2** *Ex vivo* analysis of [^68^Ga]Ga-Pentixafor and 2-[^18^F]FDG uptake into CAM-PDXs. Data are plotted as a percentage of injected dose per gram (%ID/g) and presented as mean ± SD, n = 3 per group

**Supplementary Table 2** Uptake of 2-[^18^F]FDG and [^68^Ga]Ga-Pentixafor into CAM-PDXs as assessed by *ex vivo* analysis and *in vivo* PET/MR imaging

|  | ***ex vivo* analysis** (%ID/g*) | ***in vivo* imaging** (%TA/cc†) |
| --- | --- | --- |
| [^68^Ga]Ga-Pentixafor total uptake | 11.23 ± 2.7 (n = 3) | 6.34 ± 0.35 (n = 3) |
| [^68^Ga]Ga-Pentixafor blocked uptake | 8.91 ± 2.1 (n = 3) | 5.25 (n = 1) |
| 2-[^18^F]FDG uptake | 10.51 ± 2.0 (n = 3) | 10.52 (n = 1) |

Data are presented as mean ± SD.

^*^ percentage of injected dose per gram

† percentage of total activity per cm^3^
